# Tunable structural colors on display

**DOI:** 10.1038/s41377-022-00847-z

**Published:** 2022-05-25

**Authors:** Andreas Tittl

**Affiliations:** Chair in Hybrid Nanosystems, Nanoinstitute Munich, Faculty of Physics, Ludwig-Maximilians-Universität München, Königinstraße 10, 80539 München, Germany

**Keywords:** Liquid crystals, Displays, Metamaterials

## Abstract

Structural coloration takes inspiration from the bright hues found in nature to control the reflection and transmission of light from artificially structured materials. Combining them with active electrical tuning heralds breakthrough applications in optical displays.

Producing vibrant colors has been a driving force for the development of optics, reaching as far back as the multi-colored tableaux found in the windows of medieval churches, or the distinct color-changing appearance of the Lycurgus cup from ancient Roman times. Structural coloration, where micro- and nanostructured materials are used to control the reflection and transmission of visible light at precisely tailored wavelengths, has been a cornerstone of these efforts, taking inspiration from the brilliant hues produced by, e.g., butterfly wings and macaw feathers prevalent in nature^1^. Compared to pigment- or dye-based approaches, structural coloration generally provides brigher colors with higher color gamut, improved spatial resolution, and resistance to color fading over time. A multitude of photonic concepts and systems have been implemented for this purpose^2^, ranging from thin film interference, diffraction gratings and photonic crystals^3^ to plasmonic and dielectric metasurfaces^4,5^.

Building on these advances, many important practical applications of structural coloration have been introduced including high-resolution color printing^6^, optical anticounterfeiting^7^, and colorimetric sensors^8^. The next crucial challenge for the field is the incorporation of tunability, which can unlock a new generation of planar optical devices for consumer-facing displays and other active optical components. Towards this end, a broad palette of tuning concepts has been examined, such as phase change materials, electrochromic polymers, and stretchable substrates. However, simultaneously achieving bright and tunable structural color while maintaining a dark black state has remained elusive.

Now, writing in this issue of *Light: Science & Applications*, T. Badloe and colleagues at the Pohang University of Science and Technology (POSTECH), the Sungkyunkwan University, and the National Institute of Nanomaterials Technology (NINT), Republic of Korea, have demonstrated a tunable all-dielectric metasurface satisfying these requirements, combining an array of elliptical resonators and an electrically controlled liquid crystal cell^9^. The choice of an anisotropic unit cell geometry for the metasaurface provides a dependence of the optical response on the incident linear polarization, which enables direct light modulation via the liquid crystal cell and therefore a linear transition between bright and dark states of the system (Fig. 1).

**Fig. 1:**
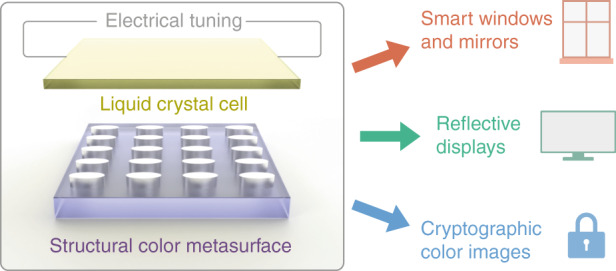
Electrically tunable structural color metasurfaces enable a multitude of applications

To optimize the color performance of the metasurface, the authors additionally leveraged the hybridization of the Mie modes of the elliptical resonators with a quasi-guided mode resonance (qGMR) of the lattice. In their analysis, the authors found that a careful tailoring of the geometric metasurface parameters (structure size, height, periodicity, etc.) was key to balance and efficiently couple the different modes in the system.

The metasurface platform developed by the authors shows striking performance, with bright red, green, and blue metasurface pixels, a color gamut comparable to the established sRGB standard, as well as high quality white and black states. These capabilities are supplemented by the continuous and fast (on millisecond timescales) modulation between states provided by the liquid crystal cell, taking crucial steps towards displays based on structural color. Such displays hold great promise for use in resource-limited settings where low power consumption is essential. However, to realize the full potential of tunable structural color for such practical applications, important constraints still need to be overcome, including the development of large-area nanofabrication for consumer-scale displays, the extension of the the color range towards wide-gamut standards, and the implementation of increased viewing angles to avoid off-axis color shifts.

These challenges open exciting perspectives for future research on structural coloring, for instance by utilizing machine learning for the inverse design of the nanophotonic geometries^10^. Finally, taking additional cues from nature can deliver a wealth of complementary functionalities for structural color materials ranging from autonomous regulation to self-healing^11^.

## References

[1] Kinoshita S, Yoshioka S (2005). Structural colors in nature: the role of regularity and irregularity in the structure. ChemPhysChem.

[2] Rezaei SD (2021). Nanophotonic structural colors. ACS Photonics.

[3] Lee HS (2013). Colloidal photonic crystals toward structural color palettes for security materials. Chem. Mater..

[4] Hedayati MK, Elbahri M (2017). Review of metasurface plasmonic structural color. Plasmonics.

[5] Yang WH (2020). All-dielectric metasurface for high-performance structural color. Nat. Commun..

[6] Flauraud V (2017). Silicon nanostructures for bright field full color prints. ACS Photonics.

[7] Hong W, Yuan ZK, Chen XD (2020). Structural color materials for optical anticounterfeiting. Small.

[8] Burgess IB, Lončar M, Aizenberg J (2013). Structural colour in colourimetric sensors and indicators. J. Mater. Chem. C..

[9] Badloe T (2022). Liquid crystal-powered Mie resonators for electrically tunable photorealistic color gradients and dark blacks. Light Sci. Appl..

[10] Huang Z, Liu X, Zang JF (2019). The inverse design of structural color using machine learning. Nanoscale.

[11] Shang LR (2019). Bio-inspired intelligent structural color materials. Mater. Horiz..

